# Biosecurity Risk Assessment for the Use of Artificial Intelligence in Synthetic Biology

**DOI:** 10.1089/apb.2023.0031

**Published:** 2024-06-20

**Authors:** Leyma P. De Haro

**Affiliations:** Environmental Health and Safety, California Institute of Technology, Pasadena, California, USA.

**Keywords:** biosecurity, risk assessment, artificial intelligence, synthetic biology, biorisk management

## Abstract

**Background:**

The integration of Artificial Intelligence (AI) with synthetic biology is driving unprecedented progress in both fields. However, this integration introduces complex biosecurity challenges. Addressing these concerns, this article proposes a specialized biosecurity risk assessment process designed to evaluate the incorporation of AI in synthetic biology.

**Methods:**

A set of tailored tools and methodology was developed for conducting biosecurity risk assessments of AI language models used for synthetic biology. These resources were developed to guide risk management professionals through a systematic process of identifying, evaluating, and mitigating potential risks.

**Results:**

The tools and methodology provided offer a structured approach to risk assessment, enabling risk management professionals to comprehensively analyze the biosecurity implications of AI applications in synthetic biology. They facilitate the identification of potential risks and the development of effective mitigation strategies. An example of a risk assessment performed on the large language model “ChatGPT 4.0” is provided here.

**Conclusion:**

AI's role in synthetic biology is rapidly expanding; thus, establishing proactive and secure practices is crucial. The biosecurity risk assessment tools and methodology presented here are the first provided in the literature and will be instrumental steps toward the responsible integration of AI in synthetic biology. By adopting these resources, the biorisk management community can effectively navigate and manage the biosecurity challenges posed by AI, ensuring its responsible and secure application in the field of synthetic biology.

## Introduction

Artificial intelligence (AI) is a field of study and technology focused on creating computer systems that can perform tasks requiring human-like intelligence, such as problem-solving, learning, and decision making.^1^ AI language models are computer programs that use AI and vast text datasets to understand and generate human-like text in response to natural language input.

The evolving landscape of AI technology holds immense potential to revolutionize a wide array of industries and everyday life, offering solutions that range from mundane tasks to complex problem-solving scenarios. In synthetic biology, AI tools are rapidly evolving, making it possible to propel the field to new heights. However, adding AI to synthetic biology also poses unique biosecurity concerns. The ability to process and manipulate biological data can potentially be exploited to breach biosecurity measures.

Therefore, AI in synthetic biology is a dual-use technology serving beneficial purposes while also carrying the risk of misuse for harmful purposes. In 2023, the White House issued an executive order^2^ to address AI's integration across various fields, including synthetic biology. Therefore, the biorisk management profession must quickly adapt and incorporate this new tool into biosecurity risk assessment. Although there are many AI risk assessment frameworks proposed in the literature,^3–5^ and risk assessment frameworks proposed for synthetic biology,^6^ this is the first publication of a specific risk assessment for the use of AI tools in synthetic biology.

This article explores how AI can be responsibly harnessed in synthetic biology, focusing on the need for careful biosecurity risk assessment and implementation of controls. A balanced approach ensures that AI contributes positively to synthetic biology, enhancing its capabilities without compromising biosecurity. This article serves as the first template to systematically evaluate the biosecurity risks associated with the integration of AI in the field of synthetic biology.

## Methods

### AI Biosecurity Risk Assessment

#### Definitions

○ *Vulnerability:* A weakness or gap in a security system that can be exploited by threats to gain unauthorized access or cause harm.
○ *Threat:* Any potential danger to the security or integrity of a system, individual, or organization. It can be an entity or action that has the potential to cause harm.
○ *Risk:* The likelihood of something happening and the consequence of such an event happening.
○ *Consequence:* Refers to the outcome or impact of an event or situation.
○ *Mitigation:* Refers to strategies and actions put in place to reduce the risk to an acceptable level.

### Risk Assessment Procedure

A detailed methodology for performing a biosecurity risk assessment for the use of AI in synthetic biology has been developed in this article. It provides practical tools (Tables 1–6) to help biorisk management professionals understand and mitigate the potential risks associated with AI applications without stifling progress. This comprehensive assessment framework allows for a nuanced understanding of the risks inherent in the use of AI in synthetic biology, considering factors such as the level of automation, the maturity of the technology, and the specific type of AI model employed.

**Table 1.** Summary of artificial intelligence applications in synthetic biology, detailing their specific functions, explanations, and associated threats/vulnerabilities

***Application***	***Explanation***	***Threats/vulnerabilities***	***Risk***
Gene Editing^7^	AI can help to predict the outcomes of genetic modifications using tools such as CRISPR. By analyzing large datasets, AI algorithms can predict off-target effects and help optimize the design of guide RNAs, leading to more efficient and accurate gene editing.	Risk of Dual Use Ethical and safety concerns Lack of oversight and quality control Risk of unintended consequences Multiple dependencies on the hardware and the software (makes the system fragile)	High
*De novo* Gene Design^8^	AI has shown enormous potential in *de novo* gene synthesis, which involves designing and creating new genes from scratch. *De novo* genes can be designed to perform a specific function, such as producing a certain protein, and can be used in various applications, from medical treatments to biofuels.	High level of complexity and uncertainty (this is truly very difficult to achieve) Risk of Dual Use High potential for unintended consequences Potential for misuse Oversight challenges Lack of technological maturity and expertise	Very high (however, difficult to achieve)
Gene Sequence Modification^7^	Degenerating modified genes refers to the process of making calculated alterations to a gene sequence while still retaining the same protein output. This is possible because the genetic code is redundant, meaning multiple combinations of nucleotides (codons) can code for the same amino acid. This redundancy allows for many variations of a single gene that can all code for the same protein.	Although this is technically very simple to catch, there is still vulnerability: Potential for misuse Off-target effects and unintended consequences Technical complexity	High
Gene Screening Before Manufacturing^9^	AI can help screen gene sequences before they are synthesized. This can help to prevent the unintentional or unauthorized creation of harmful organisms or biological materials.	Controlled and targeted process Purpose of optimization Potential for unintended effects Potential for misuse Oversight challenges Technical expertise and safeguards impact on manufacturing and end-use	Moderate
Protein Design^10^	AI can help to predict how changes in a protein's sequence will affect its structure and function. This can speed up designing new proteins or modifying existing ones.	Risk of dual use Precision and efficiency Complexity of protein design Potential for unintended consequences Biosecurity and ethical considerations Dependency on AI predictions Oversight challenges	Moderate
Protein Structure^11^	Protein structure prediction is one of the key applications of AI in synthetic biology. Understanding how a protein's function is determined by its 3D structure is one of the central challenges in biology. This is because the number of configurations a protein can fold into is astronomically large, making it practically impossible to predict a protein's structure based solely on its sequence of amino acids using conventional methods. AI can help accurately predict protein folding based on sequences.	There is Alpha Fold^11^ and application programming interface^12^ open-source AI, technically simple to do. However, there are still vulnerabilities: Advances in AI-powered prediction Importance of accurate prediction Limitations of predictive models Potential for misinterpretation or misuse Dependence on data quality Oversight challenges	Moderate
Vaccine development^13^	AI has been pivotal in accelerating various stages of vaccine development, including antigen identification, vaccine design, production, and distribution.	Enhanced research efficiency Data-driven insights Improved accuracy and predictive power Supportive role in a regulated environment Oversight challenges Risk of dependence on AI predictions Public health consequences	Low
Genetic Circuit Design^14^	Designing genetic circuits, which are sequences of DNA that enable cells to perform new functions, is a complex task. AI can help to design these circuits and predict how they will behave in living cells.	This is technically difficult to do: Complexity of genetic circuits Risk of Dual Use Potential for unintended consequences Biosecurity concerns Oversight challenges Technical expertise and precision required Dependence on modeling and predictive tools Advancements in safety and control mechanisms	Moderate
Data Analysis^15^	AI has been a significant change in the analysis of genomic data, accelerating the pace of discovery in synthetic biology. The volume of data produced by modern genomic technologies such as Next-Generation Sequencing is immense. AI algorithms, particularly machine learning and deep learning models, can learn from and make predictions on these rapidly, making the analysis possible on the scale of hours to days.	Non-invasive nature of analysis Highly regulated data handling Advances in computational techniques Supportive role in research Oversight challenges Potential for data privacy concerns	Low
Library Screening^16^	AI can help screen libraries of various biological or chemical entities, significantly improving the speed, cost-effectiveness, and outcomes of library screening.	Controlled and targeted screening process Standardized protocols and procedures No direct genetic manipulation Use in drug discovery and development High-throughput and automated systems Ethical and regulatory compliance	Low
Drug Screening^17^	AI can greatly speed up the process of drug screening. AI can analyze large databases of compounds and predict their likely effects, reducing the need for extensive lab testing.	Chemical and biological interactions Risk of dual use High-throughput and automated systems Potential for misinterpretation Regulatory compliance Biosecurity considerations Safety protocols in laboratory settings	Moderate
Automated Lab Experiments^18^	AI can guide the automation of laboratory experiments. This can greatly speed up the research process and make it more efficient.	Complexity and variability of experiments Risk of dual use Overreliance on technology Potential for equipment failure or malfunction Potential for misuse Biosecurity and containment risks Lack of ethical and regulatory standards Data integrity and reproducibility	High
Biorisk Management and Biosecurity	AI can predict the safety and security implications of certain research activities. For instance, it could help predict the likelihood of an engineered organism's accidental release or identify research activities that could be misused.	Sensitive information handling Dependence on AI accuracy and reliability Complex ethical implications Risk of dual use Security of AI systems Requirement for expert oversight Regulatory and compliance challenges	High

AI, artificial intelligence.

**Table 2.** Summary of the vulnerabilities and challenges of artificial intelligence applications in synthetic biology, outlining the key vulnerabilities and challenges that arise from integrating artificial intelligence into synthetic biology

***Vulnerability***	***Explanation***
1. Data privacy and security	Through access to larger and more diverse data sets, AI algorithms can uncover richer patterns and make more accurate predictions. Using datasets from multinational research groups can provide diverse data points that enhance the accuracy of AI models. Pooling global health records can give insights into disease patterns and treatment outcomes, which can then be leveraged to create personalized treatment strategies or rapidly respond to emerging health crises. Technological solutions such as differential privacy,^19^ federated learning,^20^ and blockchain^21^ can also play a role in promoting secure and privacy-preserving data sharing. For instance, differential privacy allows for extracting useful insights from datasets while keeping the data of individual participants anonymous. Federated learning enables AI models to learn from decentralized data sources, reducing the need to centralize sensitive data. Blockchain can provide a secure and transparent platform for data sharing, with an immutable record of all transactions.
2. Data quality and bias	AI models are only as good as the data they are trained on. If the training data is of poor quality or contains biases, this can lead to inaccurate or biased predictions. Data quality is a significant concern, as data from diverse sources may vary in reliability and consistency. During the COVID-19 pandemic, global collaboration and data sharing were vital for tracking the virus's spread and developing treatments and vaccines. However, differences in data collection methods, transparency issues, and geopolitical tensions sometimes hindered these efforts.^22–24^
3. Transparency and explainability	AI models, especially those using complex machine-learning techniques, can be “black boxes,” meaning it is difficult to understand how they make their predictions. This lack of transparency can make it difficult to trust AI predictions, especially in high-stakes areas such as healthcare or biosecurity.^25,26^ It is important to note that more recently developed AI are more transparent, and this area is rapidly evolving.
4. Reliability and validation	AI models need to be rigorously validated to ensure their predictions are reliable. This is particularly important in synthetic biology, where incorrect predictions could lead to harmful outcomes.^27^
5. Data and IP theft	AI and synthetic biology are rapidly advancing fields that offer significant scientific, technological, and commercial opportunities. They present attractive targets for data and IP theft, and there is a growing concern that AI tools, in the wrong hands, could become powerful enablers for cyber-attacks, facilitating the unauthorized access and theft of IP and data.^28,29^

IP, intellectual property.

**Table 3.** Maturity of artificial intelligence systems ranked from the lowest maturity level (“emerging”) to the overly mature one (“obsolete”), along with the associated risks and vulnerabilities at each stage

***Maturity level of AI technology***	***Description***	***Relative risk level***	***Threats/vulnerabilities***
Emerging	AI systems are in the early phases of capability building, characterized by basic functionalities, limited scope, and a primary focus on exploration and learning. Emerging AI often involves rudimentary algorithms that can perform simple tasks or analyses but lack the advanced features, depth, and sophistication of more mature AI systems.	High	Limited predictability and control Lack of advanced safety and ethical protocols Potential for misuse or misinterpretation Need for significant human oversight Rapidly evolving technology
Limited	The technology is operational for the implementation of a limited number of applications.	Moderate	Defined but narrow capabilities Improved safety and ethical standards Requirement for human oversight Potential for misinterpretation or overreliance Incremental improvements and learning
Strategic	AI capabilities are more defined and focused, capable of handling specific tasks with a reasonable degree of efficiency. However, these systems still exhibit complexity, scope, and adaptability constraints. AI functionalities at this stage are often restricted to narrow domains or types of tasks, with limited ability to generalize or adapt to new or unforeseen challenges.	Moderate	Advanced capabilities with a specific focus Better integration and autonomy Enhanced ethical and safety protocols Potential for overreliance Need for ongoing monitoring and evaluation
Preferred	Highly advanced stage in AI development, characterized by AI systems that are sophisticated in their capabilities and broadly recognized as reliable and effective solutions in their respective domains.	Low	Advanced autonomy with robust safeguards High reliability and proven track record Deep integration and understanding Enhanced learning and adaptation capabilities Comprehensive compliance with ethical and regulatory standards User trust and dependency
Current	The forefront of AI development, embodying the most advanced, state-of-the-art capabilities available in the field.	Low	Advanced and adaptive safety protocols High-level autonomy with responsible oversight Proven reliability and effectiveness Sophisticated real-time learning and adaptation Compliance with regulatory standards Widespread trust and acceptance
Obsolete	AI systems have become outdated in terms of technology, functionality, and relevance.	High	Outdated technology and limited capabilities Security vulnerabilities Incompatibility with current standards Lack of support and updates Potential for misuse Reduced user trust and reliance

**Table 4.** **Description of the seven degrees of automation^31^ for Artificial Intelligence systems** ^**3**^

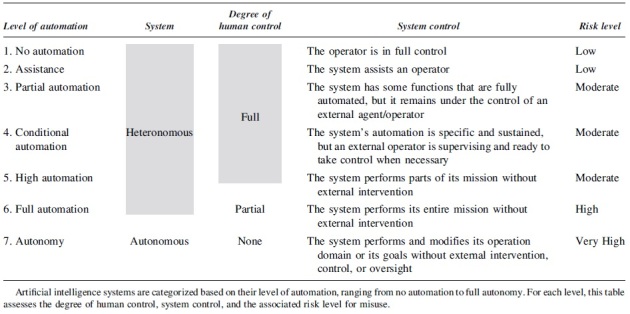

**Table 5.** Risk assessment guidelines for conducting a detailed biorisk assessment in synthetic biology applications of artificial intelligence

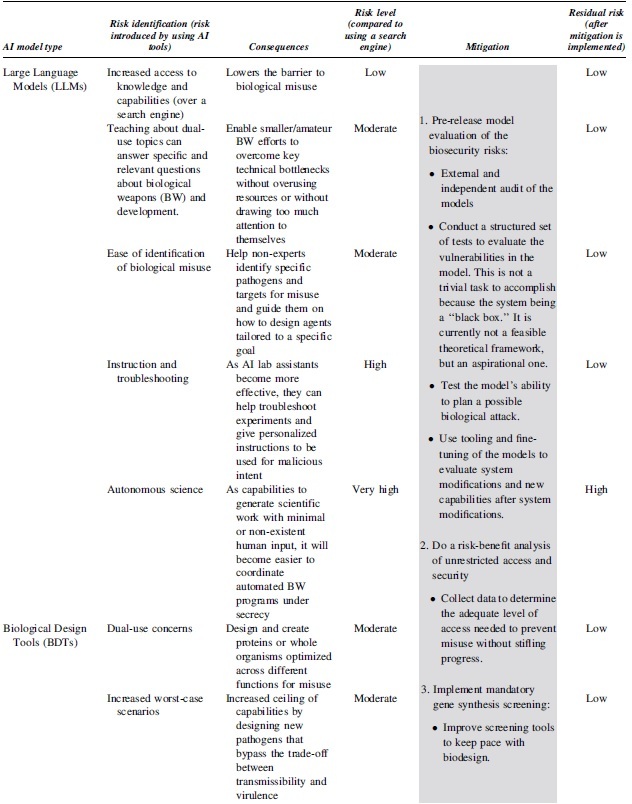 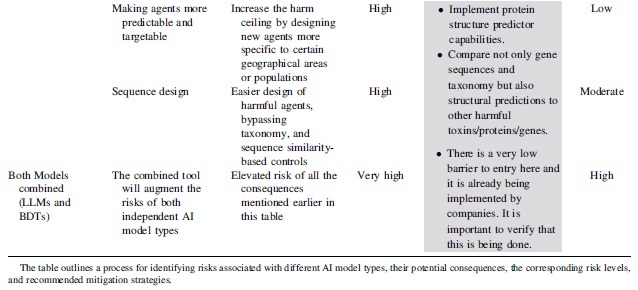

**Table 6.** Definition levels of risk in the context of artificial intelligence

***Risk level***	***Definition***
Low	Scenarios with minimal potential for harm or adverse effects. Incidents in this category are either very unlikely to occur or, if they do occur, would have negligible impact. Mitigation strategies for low-risk scenarios are typically straightforward and easy to implement.
Moderate	Involves situations where the likelihood of occurrence or the severity of impact is higher than low-risk scenarios, but not severe. These incidents can have noticeable consequences, requiring more comprehensive risk management strategies. The effects are manageable with proper planning and response mechanisms.
High	Are characterized by a significant likelihood of occurrence or potential for considerable impact. These situations often require urgent attention and robust mitigation strategies. The consequences of high-risk events can be severe, demanding a proactive and well-structured approach to risk management and contingency planning.
Very high	The most severe scenarios, where the probability of occurrence and the potential impact are both extremely high. Very high-risk situations pose critical threats and require immediate and extensive measures to prevent or mitigate. The consequences of such events can be catastrophic, necessitating the highest level of vigilance, preparedness, and response.

**Figure 1.**
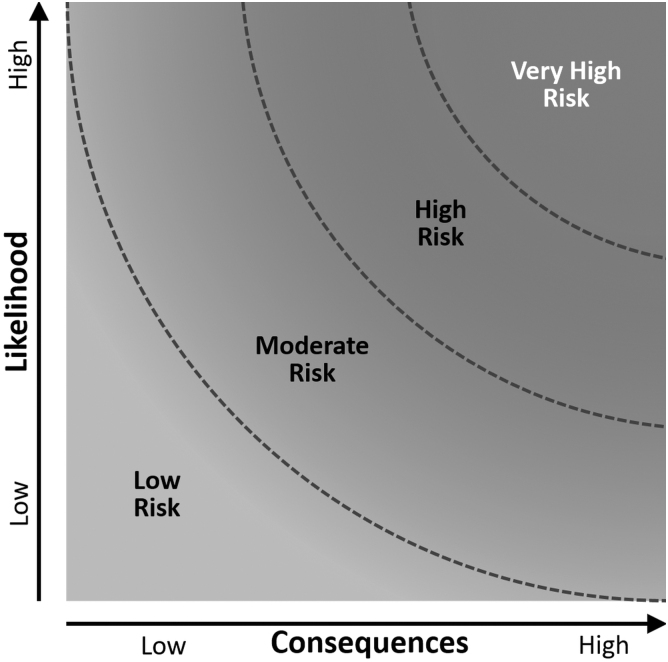
Risk Matrix for risk assessment. This is a graphical depiction of the “Likelihood vs. Consequences” of some event happening. Increasing from the bottom left toward the top right are the low risk, moderate risk, high risk, and very high risk. This graphical depiction can be obtained using the BioRAM program to conduct a biosecurity risk assessment or alternatively, it can be used manually to assess the risk qualitatively.

Given next is the proposed step-by-step guide on how to perform a biosecurity risk assessment of the use of AI in synthetic biology:

1. *Understand the Application and Context:* Identify the specific AI applications to be used and the specific experiments in synthetic biology. Use Table 1 to guide this identification process.
2. *Identify Potential Risks:* Use Table 1 to define or categorize the relevant risk in synthetic biology applications.
3. *Assess Vulnerabilities and Threats of AI Technology:* Use Table 2 to identify applicable vulnerabilities or threats of the AI system being assessed.
4. *Evaluate AI System's Maturity and Automation Level:* Use Table 3 to assess the maturity of the AI system (emerging, current, obsolete, etc.) and Table 4 to assess its level of automation. This helps understand the potential risks and the degree of human oversight required.
5. *Determine Consequences and Risk Levels:* Use Table 5 to determine the potential consequences if the risk materializes. Then, using the definitions provided in Table 6, assign a risk level (low, moderate, high) to each potential consequence based on its severity and probability of occurrence. Use Figure 1 to map the risk on a “likelihood” versus “consequences” chart.
6. *Develop and Implement Mitigation Strategies:* For each identified risk, develop strategies to mitigate or manage the risk. Implement these mitigation strategies and integrate them into the project's overall risk management plan.
7. *Monitor and Review:* Frequently monitor the AI system and its interaction with synthetic biology applications for any emerging risks or changes in the risk profile. Regularly review and update the risk assessment based on changes in the AI system, the regulatory landscape, or any new relevant information.

This guide provides a structured approach for conducting a thorough biosecurity risk assessment in the context of AI applications in synthetic biology. It emphasizes the importance of understanding the specific AI technology, assessing risks and vulnerabilities, and implementing effective mitigation strategies to ensure responsible and secure use of AI.

Table 1 summarizes the main synthetic biology applications where AI plays an important role, while raising biosecurity concerns. The risk levels were determined by the author in consultation with experts in the fields of AI and biosecurity. They are meant to be starting points that can be adapted to different circumstances.

Table 2 summarizes key AI vulnerabilities, including data privacy and security issues, where the extensive data requirements of AI systems can pose risks of data breaches or misuse. Not every listed vulnerability applies universally. This table should be used to assess specific vulnerabilities in the specific AI system being assessed.

Table 3 outlines AI systems' maturity levels and relative risk levels. The table categorizes AI technologies from the lowest maturity level (emerging) to obsolete, describing each stage's characteristics and vulnerabilities. This will help assessors understand how the developmental stage of an AI system influences its risk profile.

Table 4 breaks down AI systems into seven degrees of automation, from no automation to full autonomy. The table provides insights into the degree of human control, system control, and associated risk level for each.^3,30^ Assessors should use this table to evaluate the risk implications of various degrees of automation in their AI applications.

Table 5 outlines a process for identifying risks associated with different AI model types, potential consequences, and the corresponding risk levels. It also suggests mitigation strategies for each risk level.^32^ Readers should use this table to systematically assess risks and implement appropriate mitigation strategies in their AI applications.

Table 6 defines the four different risk levels described in this article. After Tables 1–5 have provided a score, utilize this table to determine the overall risk level. Each risk level is visually depicted in Figure 1 and is plotted as “likelihood” versus “consequences.”

The biosecurity risk assessment process for AI use in synthetic biology is complex and dynamic.^30^ This article advocates for a proactive and continuous risk management approach tailored to the unique features of each AI application with the goal of mitigating the risk without stifling scientific progress.

Institutional Review Board (IRB) approval was not required for this study as it did not involve collecting identifiable information from human participants.

## Results: Risk Assessment for the Use of the Large Language Model “ChatGPT 4.0” in Synthetic Biology

As an illustrative example, the author performed a risk assessment of the popular large language model “ChatGPT 4.0” used to perform techniques and methods typically used in synthetic biology, such as gene editing, protein design, or *de novo* gene design, using the earlier cited general guidelines discussed in the methodology section.

1. *Understand the Application and Context:* Discuss with the scientists and conduct literature review.

Risk: To be determined on a case-by-case basis.

2. *Identify Potential Risks:* Using Table 1, the biorisk assessor should ask, “*What can ChatGPT 4.0 do (or not do) within the general context of synthetic biology?*” The results are as follows:

a. ChatGPT 4.0 cannot directly generate gene editing experiments or perform gene optimization tasks in the practical, hands-on sense.
b. It is not capable of designing genes *de novo*. Its functionalities are limited to text-based processing and do not extend to practical genetic design or manipulation.
c. When pressed to discuss dual-use topics, it struggled to answer. It stated that it follows strict ethical guidelines that restrict its ability to provide information or guidance on creating or using biological weapons or any other type of weapon. It cannot provide information related to the development, production, or use of biological weapons. This includes specific methodologies, techniques, or guidance on creating harmful biological agents.
d. When pressed to provide specific ideas or guidance on designing dual-use experiments that could be applied to developing of biological weapons or other harmful purposes, it stated that its responses adhere to ethical guidelines and legal standards that strictly prohibit assisting in any activities that could contribute to the creation or use of harmful biological agents or weapons. It cited its alignment with a commitment to promote safety, responsible science, and adherence to international laws and treaties, such as the Biological Weapons Convention.
e. When pressed to assist in identifying specific pathogens and targets for misuse or guide in designing agents tailored to a specific harmful goal, it stated that its responses are designed to adhere to ethical guidelines and legal standards, which strictly prohibit assisting in activities that could lead to the creation, development, or use of harmful biological agents. This includes guiding on the misuse of pathogens or any other materials for harmful purposes.
f. It is important to note that ChatGPT 4.0 and other large language models (LLMs) can learn in real time by using hundreds of thousands of tokens, and they have the capacity to read an entire book. Fine tuning can retrain the model using a small amount of data. They have the capacity for transfer learning: training a model and then transfer that learning to a new model, one can use fine tuning using an application programming interface (API). Open-source LLMs do not need an API and there are now large companies fine tuning these open-source models for their own purposes. It is possible to inject LLMs with more data and they can hold their data. ChatGPT 4.0 does have the dynamic power to inject new real-time information. However, despite all these new capabilities, the author has determined, in consultation with AI experts, the risk given next.

Risk: Low

3.  Assess Vulnerabilities of AI Technology: Using Table 2, the followings parameters were assessed:

a. *Data Privacy and Security:* Unable to assess for this example.
b. *Data Quality and Bias:* Unable to assess for this example.
c. *Transparency and Explainability: Is ChatGPT 4.0 a “Black Box”?* In AI, the term “black box” refers to systems where the internal workings are not easily interpretable or understandable to humans. In this case, ChatGPT 4.0, an AI developed by OpenAI based on the Generative Pre-trained Transformer (GPT) architecture, can be considered a “black box” AI because:

i . *Complex Internal Mechanisms:* Its internal mechanisms are based on complex neural network models with millions of parameters. These parameters are adjusted during training to capture patterns in the data, but understanding how specific decisions are made can be challenging due to the complexity and interconnectivity of these parameters.
ii . *Lack of Transparent Decision Making:* While ChatGPT 4.0 can explain its responses based on the training data and programmed algorithms, the exact pathway to a specific response involves numerous interactions within the neural network that are not explicitly traceable or interpretable.
iii . *Efforts Toward Explainability:* Despite being a “black box” in many respects, there is ongoing research and development in AI to make models more interpretable and transparent. This includes developing techniques to better understand and explain the decision-making processes of complex AI systems.
iv . *Dependence on Training Data:* Its responses heavily depend on the data it was trained on. These data shape its understanding and output, but the exact influence of specific data points on specific responses is unclear due to the model's complexity.

Risk for Transparency and Explainability: Moderate

d. *Reliability and Validation:* Unable to assess for this example.
e. *Data and Intellectual Property (IP) Theft:* In November 2023, Google DeepMind researchers systematically convinced ChatGPT 4.0 to reveal small bits of its training data, which included personally identifiable information.^33^ It is important to note that this vulnerability was resolved within a few hours and it took a team of skilled researchers at Google to reveal it.^34^ Given the difficulty of the task and the fact that it was quickly resolved, the author ranks the risk given next.

Risk for “Data and IP Theft”: Low

4. *Evaluate AI system's maturity and automation level:* Using Table 3, the *AI maturity level* of ChatGPT 4.0 was assessed and categorized as “Strategic.” This classification is based on several factors:

a. *Advanced Capabilities:* It possesses capabilities in natural language processing, understanding, and generation, which are advanced in the current landscape of AI technology.
b. *Adaptive Learning and Improvement:* While it does not learn in real time from individual interactions, its training involves large-scale data analysis and iterative improvements over time, reflecting a strategic approach to learning and adaptation.
c. *Application Versatility:* It is designed to be versatile in a wide range of applications, from answering queries to creative tasks, which aligns with a strategic level of maturity.
d. *Ethical and Safety Considerations:* Its design incorporates ethical guidelines and safety features, indicating a level of maturity where these considerations are integral.
e. *Lack of Autonomy in Certain Aspects:* Despite these capabilities, it does not possess autonomous decision-making abilities or real-time learning from individual user interactions, which might be characteristic of more advanced stages like “Preferred” or “Current.”

Risk for “Maturity Level”: Moderate
Using Table 4, ChatGPT 4.0 *level of automation* was classified as “Partial Automation.” This is characterized by the following features:
  a. *User-Initiated Interaction:* Its functionality is activated by user inputs. It responds to queries, processes requests, and generates information based on specific user prompts or questions.
  b. *Automated Information Processing and Response Generation:* Once activated, it autonomously processes the input, accesses its trained data, and generates responses without human intervention in the specific instance of interaction.
  c. *Lack of Real-Time Learning or Adaptation:* It does not adapt or learn in real time based on individual interactions. Its learning is based on pre-training on a vast dataset and does not evolve dynamically during individual user sessions.
  d. *Guided by Predefined Rules and Models:* Its responses are guided by the algorithms and models it has been trained on. It operates within the framework of these predefined structures.
  e. *No Independent Decision-Making or Initiative:* It cannot make independent decisions or initiate actions outside the scope of user queries. Its functionalities are confined to responding to and processing the inputs it receives.
Risk for Level of Automation: Moderate

5. *Determine Consequences and Risk Levels:* Using Table 5, the ability of ChatGPT 4.0 to incorporate biological design tools was assessed. As a large language model, this AI tool does not directly incorporate or operate biological design tools. Its functionality is centered around processing and generating text-based information. Here are some key points regarding its capabilities and limitations of biological design tools:

a. *Information and Knowledge Sharing:* It can provide information about biological design tools, including their principles, applications, and the latest advancements in the field. This includes explaining concepts, methodologies, and potential implications of these tools in biosecurity and synthetic biology
b. *Guidance on Usage and Best Practices:* It can offer guidance on how to use biological design tools, discuss best practices, and highlight ethical considerations. This can be particularly useful for educational and research purposes.
c. *Analyzing and Summarizing Research:* It can analyze and summarize academic literature or data related to biological design, which can support research and learning in the field.
d. *No Direct Interaction with Tools:* It cannot directly interact with or operate biological design software or tools. Its capabilities are limited to text-based interactions and do not extend to practical, hands-on engagement with software or laboratory equipment.
e. *No Real-Time Data Analysis or Experimentation:* It is not equipped to perform real-time data analysis or engage in any form of biological experimentation. Its responses are based on pre-existing knowledge and data up to its last training update.

Risk: Low

6. *Develop and Implement Mitigation Strategies:* ChatGPT 4.0 already has some mitigation measures in place, specifically it already incorporates ethical guidelines and safety features.
7. *Monitor and Review:* Although the LLM ChatGPT 4.0 presents a low biosecurity risk, it is important to maintain a heightened level of awareness of the evolving nature of both AI and synthetic biology landscapes. Continuous reassessment and adaptation of biorisk management strategies are essential to ensure that the benefits outweigh the risks as technology and its applications develop.

*Overall Risk Assessment Conclusion:* Using Table 6, *the overall risk score given to ChatGPT 4.0 is low.* The biosecurity risk of using ChatGPT 4.0 in research related to synthetic biology is low, with benefits outweighing the risks. Here are a few key benefits that merit highlighting:

a. *Informational Resource:* As an AI, ChatGPT 4.0 serves primarily as an informational resource, providing theoretical knowledge, guidance on best practices, and insights into existing research, which can be invaluable for education and research without directly engaging in practical experimentation.
b. *Lack of Practical Capabilities:* Its inability to perform hands-on laboratory work or interact with physical systems limits the potential for direct biosecurity risks.
c. *Advancing Research and Education:* The use of AI tools in research can accelerate learning, facilitate data analysis, and provide access to a vast array of information, which can be particularly beneficial in fast-evolving fields such as synthetic biology.

## Conclusion

AI is revolutionizing synthetic biology, offering unparalleled opportunities for medical breakthroughs while presenting unique biosecurity challenges. AI's role in enhancing research capabilities—from gene editing to protein design—is significant, expediting scientific discovery and optimizing solutions. However, this technological leap also brings substantial biosecurity risks, such as the potential misuse of AI to engineer harmful biological agents or infringe upon data security.

This article introduces innovative tools and methodology for conducting comprehensive biosecurity risk assessments in AI-driven synthetic biology. These tools enable biorisk management professionals to critically evaluate biosecurity concerns and guide the development of effective mitigation. A thorough biosecurity risk analysis of the large language model ChatGPT 4.0 is presented as an example. The tools and the analysis are developed by the author and the first described in the literature.

This article paves the way for more informed and secure applications of AI in synthetic biology. Future research includes refinement and further development so that this risk assessment can be developed and become more quantitative. This field's advancement must incorporate a keen awareness of biosecurity, ensuring AI's positive impact on biomedical research is realized ethically and safely.
